# Transcriptome-wide association studies implicate *RCC1* and *PHACTR4* in prostate cancer survival

**DOI:** 10.1038/s41431-026-02147-1

**Published:** 2026-06-20

**Authors:** Weijia Fu, Joseph H. Rothstein, Olle Melander, Hans Lilja, Weiva Sieh, Xiaoyu Song, Robert J. Klein

**Affiliations:** 1 Tisch Cancer Institute, Icahn School of Medicine at Mount Sinai, New York, NY USA; 2 Department of Genetics and Genomic Sciences, Icahn School of Medicine at Mount Sinai, New York, NY USA; 3 Department of Epidemiology, MD Anderson Cancer Center, Houston, TX USA; 4 Department of Clinical Sciences, Lund University, Malmö, Sweden; 5 Department of Internal Medicine, Skåne University Hospital, Malmö, Sweden; 6 Departments of Pathology and Laboratory Medicine, Surgery (Urology Service), and Medicine (GU-Oncology Service), Memorial Sloan Kettering Cancer Center, New York, NY USA; 7 Department of Translational Medicine, Lund University, Malmö, Sweden; 8 Centre for Biomedical Data Science, Duke-NUS Medical School, National University of, Singapore, Singapore

**Keywords:** Prostate cancer, Genome-wide association studies

## Abstract

While prostate cancer (PrCa) is highly heritable, the genes associated with PrCa survival after diagnosis remain poorly understood. We aimed to identify genes associated with PrCa-specific survival through transcriptome-wide association studies (TWAS) using genetic predictors of gene expression in the prostate. We used the Transcriptome-Integrated Genetic Association Resource (TIGAR) to train expression prediction models separately using normal prostate, primary tumor, and metastatic tumor tissues. We performed TWAS using these models in data from the Malmö Diet and Cancer (MDC) study (discovery) and Prostate, Lung, Colorectal, and Ovarian (PLCO) Cancer Screening Trial (validation). We identified and validated seven genes associated with PrCa-specific survival at a common locus on chromosome 1. We found two genes using prediction models from normal prostate tissues and five with models from metastatic tumor tissues. Elevated *RCC1* expression is linked to a shorter time to biochemical recurrence, while higher *PHACTR4* expression was observed in tumors with a higher Gleason grade. The study is limited to European ancestry and can only show associations. Further research across other populations and experiments to establish causality will be needed. Our multi-tissue study identified novel genes associated with PrCa survival, particularly *RCC1* and *PHACTR4*, providing new insights for potential genomic markers for PrCa survival.

## Introduction

Prostate cancer (PrCa) ranks as the second most common cause of cancer-related deaths among American men, yet most diagnosed cases are not fatal [1]. The seemingly contradictory situation arises from the slow-growing nature of most prostate cancers and the presence of cancer cells in the prostate gland in many men at older age [2]. While many of these cancers do not progress to pose risk to quality or length of life, a minority of tumors progress and form metastatic lesions, requiring aggressive treatments, and cause significant morbidity and potentially death from the disease. These issues have clinical implications for prostate cancer screening with respect to optimizing the balance of benefits of decreased disease mortality through early detection with harms of overdiagnosis and overtreatment [3, 4].

PrCa is strongly heritable [5, 6]. This observation has led to numerous genomic studies aimed at identifying constitutive genetic variants that could enhance the identification of men at a higher risk for prostate cancer. These studies have yielded significant results. A recent genetic study identified over 451 distinct variants affecting the risk of incident prostate cancer diagnosis [7]. Analysis of polygenic risk scores based on these variants found that men in the top decile of risk are 5 times more likely to be diagnosed relative to the median [7]. Transcriptome-wide association studies (TWAS) using gene expression data from normal prostate tissue samples revealed 38 replicated genes regulated by germline variants with putative effects on PrCa risk [8].

However, the PrCa-risk associated variants and genes don’t necessarily correlate with disease-specific survival after diagnosis [9, 10]. While prostate cancer outcomes, similar to risk, exhibit familial clustering, implying a heritable aspect [11, 12], the influence of inherited genetic variations and their responsible genes on prostate cancer outcomes in diagnosed men are less studied. In a recent genome-wide association study (GWAS), we found multiple loci associated with survival time [13], including significant associations at the *AOX1* and *SMG7* genes. However, this study was limited by its exclusive examination of unscreened Swedish men and did not include a transcriptome-wide approach for gene identification.

Here, we conducted comprehensive transcriptome-wide association studies on PrCa-specific survival in diagnosed patients to distinguish between lethal and indolent PrCa. We first trained the genetic imputation models of expression for European ancestry (EA) men on three tissue types, including normal prostate, primary prostate tumor, and metastatic prostate tumor. Next, to identify PrCa survival-associated genes in different disease stages, we applied these models for transcriptome-wide association studies using Malmö Diet and Cancer (MDC) GWAS summary statistics derived from PrCa-specific survival analysis of 1053 patients of whom 245 have died of prostate cancer [13]. Then, we validated significant genes utilizing data from 5385 PrCa-diagnosed patients from Prostate, Lung, Colorectal, and Ovarian (PLCO) Cancer Screening Trial, of whom 274 have died of prostate cancer. These analyses identified multiple significant genes at a common locus on chromosome 1p35.3.

## Methods

### Building prediction models

#### Normal prostate tissue

We built gene expression prediction models using Genotype-Tissue Expression (GTEx) Project [14], v8 genotype and gene expression data for normal prostate tissues from 183 European ancestry (EA) men who died of other causes, though we cannot discount the possibility of undiagnosed prostate cancer in their prostates (dbGap accession number phs000424.v8.p2). The EA was determined as self-reported non-Hispanic white in their phenotype data. Following the protocols of GTEx consortium [14], we selected genes with expression thresholds of >0.1 TPM (transcript per million) in at least 20% of samples and ≥6 reads in at least 20% of samples, resulting in a set of 26,585 out of 56,200 genes. Expression data were normalized between samples using TMM in edgeR [15] and then further normalized across samples using a quantile normal transform. The genotype data was obtained from whole genome sequencing (WGS). To boost identifications in the downstream association analysis, we filtered single nucleiotide polymorphisms (SNPs) in 1000 Genome and with minor allele frequency <1% in our data. We also removed all indels, monomorphisms, and ambiguous pairs (e.g. A/T, C/G), SNPs with >20% missing genotypes, and SNPs with Hardy–Weinberg equilibrium (HWE) test *p*-value < 1 × 10^−5^. To build the model, we included all SNPs located from 1 Mb upstream of the transcriptional start to 1 Mb downstream of the transcriptional end. This includes all intronic and exonic SNPs in the transcribed region plus those intergenic regions within 1 Mb of the gene.

To train the prediction models, we used the Transcriptome-Integrated Genetic Association Resource (TIGAR) [16] approach. TIGAR is a nonparametric method based on Bayesian Dirichlet Process Regression that demonstrated boosted prediction accuracy from parametric methods like PrediXcan. TIGAR is actively maintained and thus can be used to train comparable models in normal, primary tumor, and metastasis tumor samples. Covariates adjusted in model training included genomic principal components (PC) 1–5, PEER factors 1–30, PCR, platform, age, and RIN number. Imputation R^2^ was generated by 5-fold cross-validation (CV). To assess if the Bayesian models generated by TIGAR predict gene expression better than the elastic net models built with PrediXcan, we also trained models using the same reference genetic/gene expression data and the implementation of the PrediXcan algorithm in TIGAR.

#### Primary prostate tumors

To investigate the PrCa-survival associated genes expressed in primary tumors, we also built gene expression prediction models using the Cancer Genome Atlas (TCGA) [17] genotype and gene expression data for primary prostate tumors from 373 EA men (dbGap accession number phs000178.v11.p8). We downloaded the raw CEL files for the Affymetrix SNP 6.0 arrays from adjacent normal tissue from the legacy TCGA data portal and called genotypes using the *apt-probeset-genotype* command line tool from Affymetrix. We removed all indels, monomorphs, ambiguous allele pairs (e.g. A/T, C/G), SNPs with >5% missing genotypes, and HWE test *p*-value < 1 × 10^−5^. The remaining SNPs were aligned to build 38 coordinates, and imputation was performed on the TOPMed imputation server [18]. Then we used this processed genotype data to determine the ancestry by performing principal component analysis (PCA) using the FastPCA approximation in EIGENSOFT v6.1.4. From this, we identified a total of 373 men who appear to be of European ancestry for building the gene expression prediction models. We processed the expression data in the same manner as we did for GTEx, resulting in 22,474 out of 55,325 genes. The prediction models were trained using TIGAR for these 22,474 genes using SNPs within ±1 Mb of the gene start and end locations with imputation score >0.7, appearing in 1000 Genome, and with minor allele frequency >1% in our data. Covariates included PCs 1–10 and age.

#### Metastatic prostate tumors

To investigate the PrCa-survival associated genes expressed in metastatic PrCa, we built genetic prediction models using data from the Genomic Characterization of Metastatic Castration Resistant Prostate Cancer (GCMCRPC) study [19]. This study had WGS data and RNA-seq data from 99 subjects; of these 84 were of self-reported white race. The vast majority of samples come from either lymph node or bone metastases, though some come from other sites [19]. We followed the same protocols as GTEx for preprocessing the WGS and gene expression data, resulting in 28,472 out of 60,483 genes for model training. We trained the TIGAR prediction models for these 28,472 genes using SNPs within ±1 Mb of the gene start and end locations, using PCs 1–10, site of resection or biopsy, and hidden expression covariates 1–10 as covariates. Hidden covariates were calculated following the method by Mostafavi et al. [20], using gene expression data and site of biopsy.

### Transcriptome-wide association study using MDC GWAS summary statistics

We had previously reported a GWAS of prostate cancer survival in the MDC cohort [13] which consists of 1053 men with prostate cancer (European ancestry, white), aged 45–73, in Malmö, Sweden. Of these, 245 men have died from prostate cancer after follow-up for 22 years. The summary data contained statistics for 6,246,818 variants for their association with PrCa survival, covering 62.5%, 67.2%, and 60.2% of the SNPs used in gene expression training model of GTEx, TCGA, and GCMCRPC datasets respectively. We calculated linkage disequilibrium (LD) for these SNPs using reference LD genotype covariance file generated separately from GTEx, TCGA and GCMCRPC datasets. The genome segmentation block used for LD calculation was ±1 Mb from the gene, consistent with the gene region used in predictive model training for gene expression. TWAS was conducted separately for normal prostate, primary PrCa tumor, and metastatic PrCa tumor tissues. For each gene in each data, we used TIGAR SNP weights, LD calculated from corresponding dataset, and GWAS summary Z-score statistics from MDC to obtain the test Z-score and p-value for gene expression associations with PrCa-specific survival using the S-PrediXcan strategy [21] as implemented in the TIGAR software. The Z-score statistics from the MDC come from our prior Cox proportional hazards survival analysis of the MDC data [13]. We calculated the false discovery rate (FDR) using the Benjamini–Hochberg procedure [22] and family-wise error rate (FWER) using the Bonferroni correction for the number genes tested in each tissue type.

### Validation of TWAS hits in PLCO cohort

The subjects were genotyped on five different microarrays. For each array, only single nucleotide variants with unambiguous strand alignment, a missingness rate <5%, and a Hardy–Weinberg equilibrium *p*-value > 10^–5^ were considered. We processed the genotype data with a custom in-house script that converted all data to genome build 38. To do this, we first created a master list of mappings between genome build 37 and genome build 38 by running liftOver repeatedly for each possible chromosome position. We then used this table to map each SNP from build 37 to 38, taking into consideration the portion of the genome whose orientation flipped between the two builds. This step was necessary to ensure that these regions do not fail QC on the imputation server [23]. The resulting VCF files were then imputed to the TopMed imputation server using Eagle phasing [24–26]. Data was imputed as one batch per array except for the GSA array, which we split into four random sets due to limitations of the imputation server. To estimate the principal components of genetic ancestry, we used the build 38-aligned files prior to uploading to the imputation server. We merged the data population reference data [27, 28] lifted over to build 38. Variants with missingness less than 0.1% were then LD pruned and principal components were calculated using the “fast” mode of the smartpca program in EIGENSOFT [29]. To run the survival analysis, we extracted only those individuals with a prostate cancer diagnosis and only those imputed variants with r^2^ > 0.8.

As the majority PrCa patients die from other causes than the cancer, we modeled death of other causes (DoO) separately from death of disease (DoD) using competing risk models. Specifically, we first predicted the genetically regulated gene expression (GReX) for each significant genes with FDR < 10% in MDC, using TIGAR models trained on normal, primary tumor, and metastasis tumor tissues. Next, we leveraged a multi-state R package *mstate* [30] to perform competing risk modeling. To use the multi-state method, we defined three disease stages, PrCa diagnosis (stage 1), DoO (stage 2), and DoD (stage 3), and their transition probabilities. We required the transition probabilities to be nonzero for staying in the same stage (e.g. stage 1 → stage 1), or transiting from diagnosis to DoO (stage 1 → stage 2), or transiting from diagnosis to DoD (stage 1 → stage 3), but to be zero for the reverse of the transition or transition between stage 2 and 3. Then, we fitted the multi-state model between the imputed GReX and the three disease stages, adjusting for the top 10 genetic PCs, age at diagnosis, aggressive prostate cancer (Gleason ≥ 8), and advanced cancer (T stage ≥ 3 or M stage = 1). This analysis provided log hazard ratios for each gene on DoD. Genes that were significant at a nominal level of *p* < 0.05 and exhibited the same association direction as in MDC cohort were considered validated.

### Colocalization analysis

To test if the same genetic variant is driving associations with gene expression changes and prostate cancer survival, we performed colocalization analysis using the coloc package [31]. This package first fine maps the signal for each trait (gene expression and survival) using Susie [32] and then uses a Bayesian approach to determine the posterior probability that the same variant is driving the association with both traits, taking linkage disequilibrium into account. To perform coloc analysis, for each identified TWAS hit we included all SNPs from the TIGAR expression imputation model and compared the association with gene expression from the training data with the association signal from the MDC cohort.

### Association between gene expression and time to biochemical recurrence

To understand the effects of validated genes on the time to PrCa biochemical recurrence, we used measured transcriptomics data from three independent cohorts available in the Cambridge Carcinoma of the Prostate App [33]. These cohorts are referred to by their geographic origin – MSKCC [34], Stockholm, and Cambridge [35]. We performed Cox regression to assess the association between gene expression and time to biochemical recurrence for all genes validated in PLCO, adjusting for Gleason score (≤7 and ≥8), clinical stage (T1, T2 and T3, T4), PSA category (<10, 10–20, >20), and age at diagnosis (not in Stockholm cohort). .We then combined the results for each gene across three cohorts using random effects meta-analysis in R package metafor [36]. As a sensitivity analysis, we altered the categorization of covariates as: Gleason ≤6 vs. Gleason ≥7, T1/2 vs T3/4, PSA < 10 vs. PSA ≥ 10, and age at diagnosis <60 vs. ≥60.

### Association between gene expression and tissue types in TCGA data

To understand the functional roles of significant genes, we compared the gene expression levels of all validated genes first between 43 tumor adjacent normal prostate tissues and 375 tumor tissues, and then between 208 low grade primary tumor and 167 high-grade primary tumor tissues in TCGA. Normalization was performed on the combined expression data from both normal and tumor tissues using TMM and then quantile normal transform as done for the TWAS model building. We compared tumors with Gleason score ≤7 to those with Gleason ≥8. Two-sided Student’s *t* test was used for the comparison. As a sensitivity analysis, we also compared Gleason 6 with Gleason ≥7, and Gleason 6 with Gleason ≥8.

## Results

### TWAS models in normal, primary tumor and metastatic prostate tissues

**Fig. 1:**
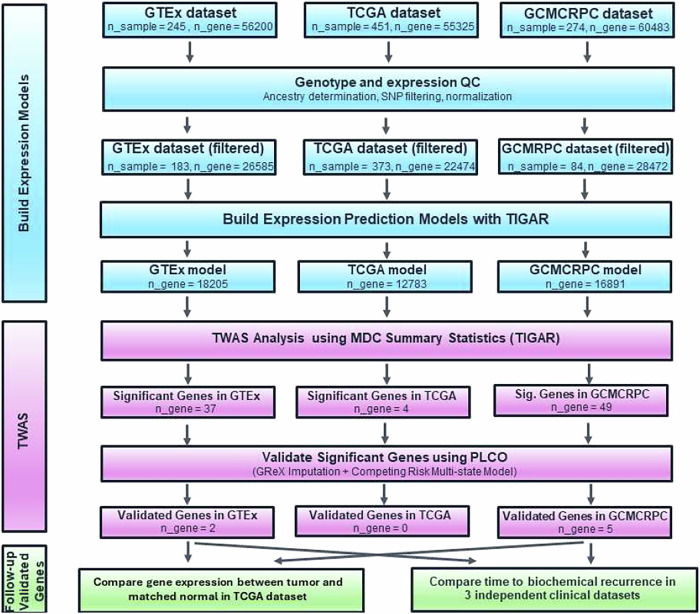
Study design. This flowchart illustrates how data moves through the study, from generating the gene prediction models (blue) to applying them in the TWAS (purple) to confirmatory analyses (green).

An outline of the study design is shown in Fig. 1. To capture critical genes for PrCa-specific survival across different disease stages, we built the genetic prediction models of expression on normal prostate tissue samples (*n* = 183), primary prostate tumor tissue (*n* = 373), and metastatic prostate tumor tissues (*n* = 84), all from men of European ancestry (EA). Genes with CV R^2^ > 0.005 [16] were included in the TWAS (Table 1), which represented 18,205 (68%) genes in normal prostate tissue, 12,783 (57%) genes in primary tumor tissue, and 16,891 (59%) genes in metastatic tumor tissue. We compared these results to the CV R^2^ observed if we trained models using an elastic net approach as in PrediXcan, using the same reference data. Across all three tissue-specific models, the elastic net approach consistently yielded a lower proportion of genes with CV R² > 0.005 and a higher proportion of genes with CV R² < 0.005 compared to the Bayesian model (Table 1; Supplementary Table S2). We also asked how well each of the three models could predict gene expression in the other tissue types. We noted that while self-prediction almost always was more accurate than prediction in a different tissue type, for most genes the predictive models could also predict expression with similar mean square error (MSE) in the other tissue types (Supplementary Fig. S2).

**Table 1:** Cross-validated R^2^ for prostate gene expression prediction models, trained in GTEx normal, TCGA tumor, and GCMCRPC metastasis samples using the Bayesian framework of TIGAR.

CVR^2^	GTEx (Normal)	TCGA (Primary Tumor)	GCMCRPC (Metastasis)
<0.005	8380 (32%)	9691 (43%)	11,581 (41%)
[0.005, 0.01)	1396 (5%)	3544 (16%)	321 (1%)
[0.01, 0.05)	11,793 (44%)	8301 (37%)	7241 (25%)
[0.05–0.1)	3037 (11%)	491 (2%)	6613 (23%)
≥0.1	1979 (7%)	447 (2%)	2716 (10%)
Total	26,585	22474	28472

### TWAS identified genes associated with PrCa-specific survival across all three tissue types

**Fig. 2:**
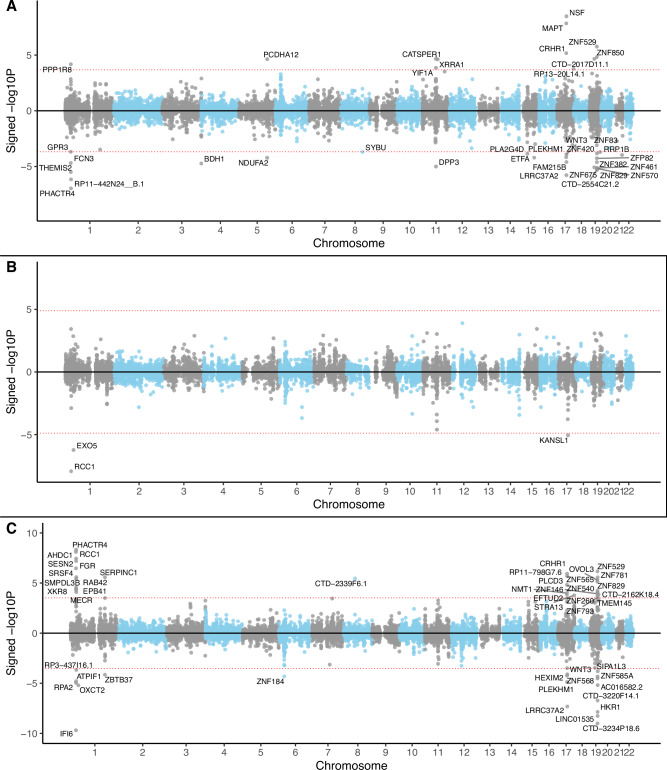
Miami plot of TWAS of PrCa-specific survival in MDC cohort. TWAS was performed using gene expression imputation models trained on different prostate datasets. **A** GTEx normal. **B** TCGA tumor. **C** GCMCRPC metastases.

We performed TWAS using these genetic prediction models on our previously published GWAS summary statistics of PrCa-specific survival [13]. The summary statistics included 6,246,818 SNPs derived from 1053 diagnosed EA patients, of whom 245 died of the disease. The genome inflation factors estimated using the Bayesian method BACON [37] were 0.84 for normal prostate, 0.84 for primary tumor, and 0.80 for metastatic tumor (Supplementary Fig. S1), indicating no inflated type I error in all three tissue types. At 10% FDR, we identified 37 genes in normal prostate, 4 genes in primary tumor, and 49 genes in metastasis tumor associated with PrCa-specific survival (Fig. 2 and Supplementary Table S1). Using Bonferroni correction (*p* < 4.01e−06), we identified 6 genes in normal prostate, 2 genes in primary tumor, and 10 genes in metastatic tumor associated with PrCa-specific survival (Fig. 2 and Supplementary Table S1).

The choice of CV R^2^ cutoff for including a gene can influence the result in two ways. Including more genes allows for potential discovery of associations with those additional genes, at the expense of a stricter multiple testing correction. To explore the effect of a stricter CV R² threshold, we increased it from 0.005 to 0.01 and recalculated the FDR. The number of significant genes identified in the GTEx discovery cohort decreased slightly from 37 to 34, while the counts for TCGA and GCMCRPC remained unchanged. The number of validated genes in all three tissues remained the same. Overall, these results suggest that changing the CV R² threshold has only a minor impact on the detection of significant genes.

### Significant PrCa-survival associated genes are validated in PLCO

To validate the significant genes in an independent cohort, we analyzed data from the PLCO screening trial, which consists of 5385 PrCa patients of EA with 274 having PrCa-related death. Seven genes from two tissue types that were significant at *p* < 0.05 and exhibited the same association direction as in MDC cohort were considered validated (Table 2), including *PHACTR4* and *PPP1R8* in the normal tissue and *ATPIF1*, *MECR*, *RCC1*, *RP3-437I16.1* and *SRSF4* in metastases. Notably, all validated genes were from the same locus on chromosome 1, suggesting that the significant results for all these genes may be due to pleiotropy, in which the same genetic variants are associated with expression levels of numerous genes as well as PrCa-specific survival. We noted that while the observed and predicted expression levels of these genes were correlated, not all genes in this region were significant in the TWAS (Supplementary Figs. S3, S4).

**Table 2:** TWAS results for validated genes.

Dataset	Gene Name	CHROM	Start	End	No of SNPs	CV R2	MDC Z score	MDC *P*-value	PLCO Z score	PLCO *P*-value
GTEx	PPP1R8	1	27830778	27851676	1740	0.054	3.975	7.05E−05	1.976	4.80E−02
GCMCRPC	ATPIF1	1	28236109	28246906	2006	0.032	−4.308	1.65E−05	−2.202	2.77E−02
GTEx	PHACTR4	1	28369603	28500369	2244	0.043	−5.315	1.06E−07	−2.321	2.00E−02
GCMCRPC	RCC1	1	28505943	28539300	2334	0.053	5.506	3.67E−08	2.419	1.56E−02
GCMCRPC	SRSF4	1	29147743	29181987	3282	0.055	4.698	2.62E−06	2.039	4.15E−02
GCMCRPC	MECR	1	29192873	29230942	3381	0.030	3.939	8.19E−05	2.814	4.90E−03
GCMCRPC	RP3-437I16.1	1	29329620	29350114	3456	0.038	−3.695	2.20E−04	−2.255	2.41E−02

In an attempt to further narrow down potentially causal genes, we asked if there was evidence for the same genetic variant driving both the association with gene expression and prostate cancer survival using colocalization analysis [31]. We did not find any gene for which the posterior probability of the same genetic signal driving both associations was high (Supplementary Table S3). Notably, we found hardly any genes for which the posterior probability of two separate genetic variants driving the two phenotypes was observed. This emphasizes the limited power of colocalization analysis and does not allow us to further narrow down the list of potentially causal genes.

### Expression level of RCC1 was associated with shorter time to biochemical recurrence

**Fig. 3:**
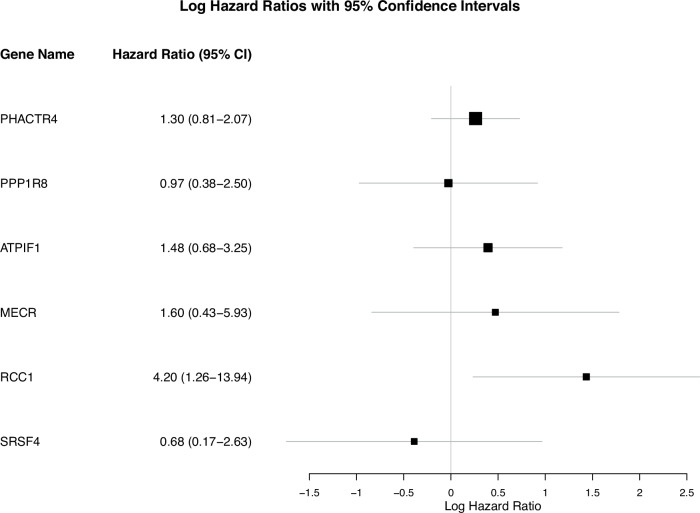
Log hazard ratios and 95% confidence intervals for validated genes. Points represent estimated log hazard ratios from the Cox regression model, and horizontal lines indicate the corresponding 95% confidence intervals. The data comes from 343 total patients in the Cambridge Carcinoma of the Prostate App [33].

Assuming shared genetic effects on chromosome 1 would make it difficult to disentangle causal genes from genetically correlated bystanders for PrCa-specific survival, we next examined the directly measured gene expression levels in three published cohorts [33–35] for their effects on time to biochemical recurrence through a meta-analysis. We found that increased expression levels of *RCC1* in primary tumors is associated with shorter time to biochemical recurrence (*p* = 0.019; Fig. 3), consistent with TWAS results. This association remained significant in the sensitivity analysis where we tried different categorizations for the covariates (all *p* < 0.05). We also observed *MECR* was the only other gene associated in a consistent direction with the TWAS results, though it was not statistically significant.

### Six validated genes were associated with tumor-normal difference and two were associated with tumor grade

**Fig. 4:**
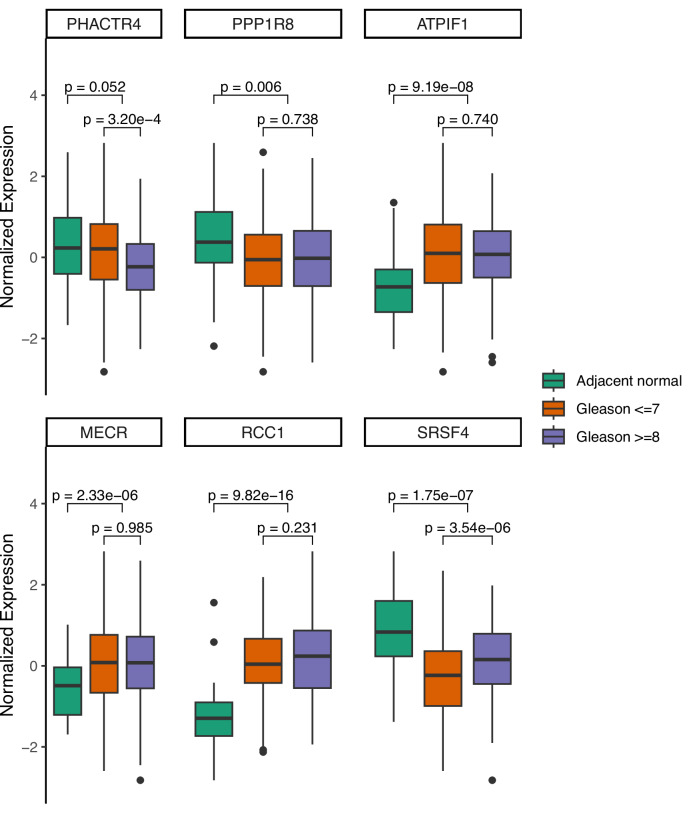
Comparison of gene expression across prostate tumor-adjacent normal, low-grade prostate tumor, and high-grade prostate tumor in TCGA data. *P*-values were calculated using two-sided Student’s *t* test to compare gene expression between adjacent normal and tumor tissues, as well as between tumor tissues with Gleason scores ≤7 and those with Gleason scores ≥8.

To understand if the validated genes were differentially expressed between prostate tumors and adjacent normal tissue, we analyzed RNA-Seq data from TCGA [17]. We found that all six genes found in the TWAS that were present in TCGA showed a difference in expression between tumor and normal (Fig. 4). The smallest *p*-value was observed for *RCC1*, for which higher expression was observed in tumors.

To understand if these genes were differentially expressed by grade, we also compared tumors with Gleason score ≤7 to those with Gleason ≥8 in TCGA (Fig. 4). Significant differences were observed for *PHACTR4* and *SRSF4*. As higher Gleason grade was associated with worse survival, the direction of effect for both genes was consistent with the observed effect of imputed gene expression on survival from the TWAS. However, in a sensitivity analysis that compared Gleason 6 tumors to those with Gleason either ≥7 or ≥8, a significant difference in expression was not observed for these two genes.

## Discussion

In this study, we conducted TWAS to identify PrCa survival-associated genes. We first trained gene expression prediction models among EA men on normal, primary tumor, and metastatic tumor tissues separately. These models were then applied to our previous GWAS data to identify genes for which imputed expression at various disease stages was associated with PrCa survival. Significant genes were subsequently validated using data from the PLCO study. We found two genes in normal and five genes in metastatic tumor tissues whose association with PrCa survival was validated in the PLCO. Among these seven validated genes, *RCC1* was associated with shorter time to biochemical recurrence; six were associated with tumor-normal difference; and two (*PHACTR4* and *SRSF4*) were associated with tumor grade.

Our study makes unique contributions to the field of prostate cancer research by addressing the challenges of acquiring long-term survival data for prostate cancer patients. While previous TWAS have focused on risk of incident diagnosis of prostate cancer, our study is among the first to employ TWAS specifically to identify genes associated with prostate cancer survival. Additionally, our comprehensive analysis across three tissue types provides a unique perspective on how gene expression patterns in distinct stages of the disease may impact survival that single-tissue studies cannot capture.

The existing literature is consistent with the hypotheses that increased levels of *RCC1* and decreased levels of *PHACTR4* lead to shorter survival times in prostate cancer. *RCC1* is a guanine nucleotide exchange factor for the Ras paralog Ran and plays a role in both nuclear transport and mitotic spindle assembly [38]. It has been shown to play a role in the cell cycle and DNA repair [39]. Intriguingly, Ran has been posited to mediate the androgen-independent transport of the androgen receptor to the nucleus, suggesting a role in androgen-independent prostate cancer [40]. Using a shRNA screen in human mammary epithelial cells, *PHACTR4* was found to be a tumor suppressor [41]. It binds with protein phosphatse1 (PP1), thereby preventing PP1 from dephosphorylating and activating the tumor suppressor Rb [41, 42]. It also can function in an Rb-independent manner [41], including by inhibiting the IL-6/STAT3 pathway [43]. While our TWAS data cannot distinguish between these two hypotheses, the associations of gene expression with biochemical recurrence [14] argues in favor of *RCC1* being responsible for the observed association between SNPs at this locus and prostate cancer survival (Fig. 2). Further research will be needed to elucidate the mechanism behind this association.

Several limitations to this study should be noted. First, the TWAS approach only identifies association and cannot demonstrate causation. Additional work, including biological experiments, will be needed to establish causality. Similarly, the TWAS approach alone cannot distinguish between several genes associated at the same locus. While the orthogonal evidence provided by the association of gene expression with biochemical recurrence suggests *RCC1* may be the key driver of the association signal, further work will be needed. For building gene expression prediction models for metastatic tumors, we had included data from different metastatic sites (e.g. bone, lymph nodes) as there were not enough subjects with biopsy taken from the same metastatic site. We also only considered prostate-derived tissue in building our gene expression models. Gene expression changes in other tissues could influence survival through various mechanisms, and would not be captured by this study. Including other tissues, or performing cross-tissue TWAS, could enable further gene discovery. This study did not consider the effects of treatment, as treatment data was not readily available in the MDC study; further studies focusing on survival genetics in cohorts with high quality treatment information is warranted. Finally, this work was restricted to individuals of European ancestry. It remains to be seen whether similar associations hold in other populations, or if other associations can be found with TWAS in different populations.

## Supplementary information

Supplementary Figures and Supplentary Table S2

Supplemental Tables S1 and S3

## Data Availability

The underlying data to build the gene expression prediction models is available from dbGaP under the accession numbers specified in the acknowledgements. Data from the PLCO trial is available from dbGaP and from NIH’s Cancer Data Access System (http://cdas.cancer.gov/). Our trained gene expression prediction models for GTEx and the gene-level summary statistics for the TWAS using all prediction models are available from Zenodo under DOI 10.5281/zenodo.19892434. We cannot make the models from TCGA and GCMCRPC available as dbGaP has not given permission to share summary statistics from those studies.
