# Supplementary material for: Transcriptome-wide association studies implicate RCC1 and PHACTR4 in prostate cancer survival
Source: Eur J Hum Genet. 2026 Jun 20;34(8):1147–55. doi: 10.1038/s41431-026-02147-1 (PMC13424128; doi:10.1038/s41431-026-02147-1)
Supplement: Supplementary file 1 — Supplementary Figures and Supplentary Table S2 [file 41431_2026_2147_MOESM1_ESM.docx]

**Supplementary Materials**

Figure S1: QQ plot for MDC cohort

Figure S2. Scatterplots comparing the mean squared error (MSE) of gene expression prediction models when applied to the training dataset and to other datasets. A) Model trained on GTEx predicting expression in GTEx vs TCGA. B) Model trained on GTEx predicting expression in GTEx vs metastases. C) Model trained on TCGA predicting expression in TCGA vs. GTEx. D) Model trained on TCGA predicting expression in TCGA vs. metastases. E) Model trained on metastases predicting expression in metastases vs. GTEx. F) Model trained on metastases predicting expression in metastases vs. TCGA

1. B)


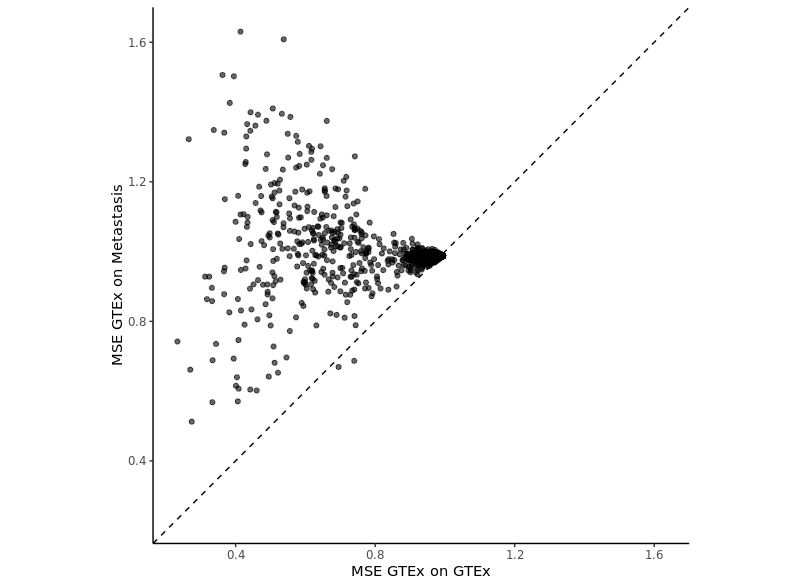


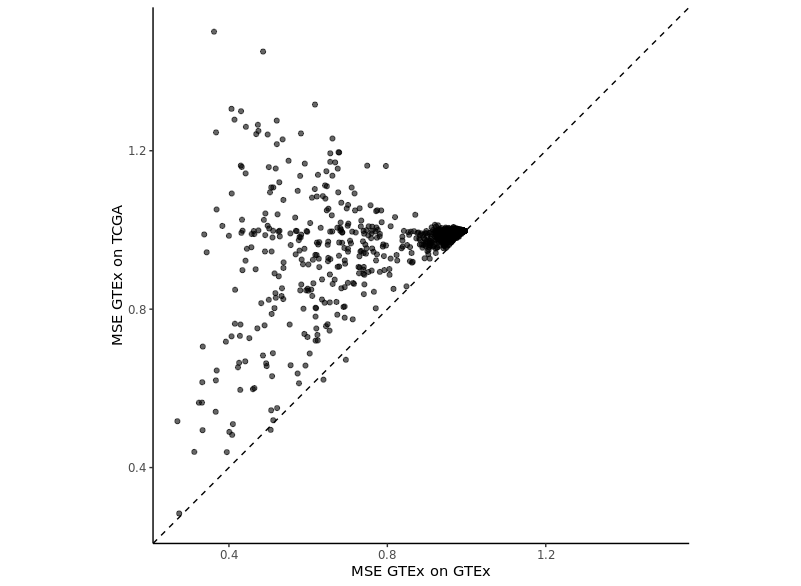


C) D)

**
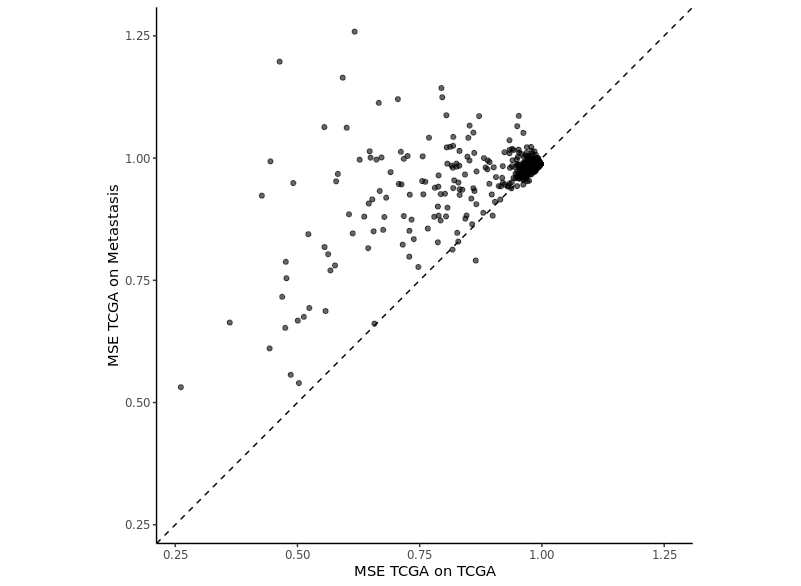
**

**
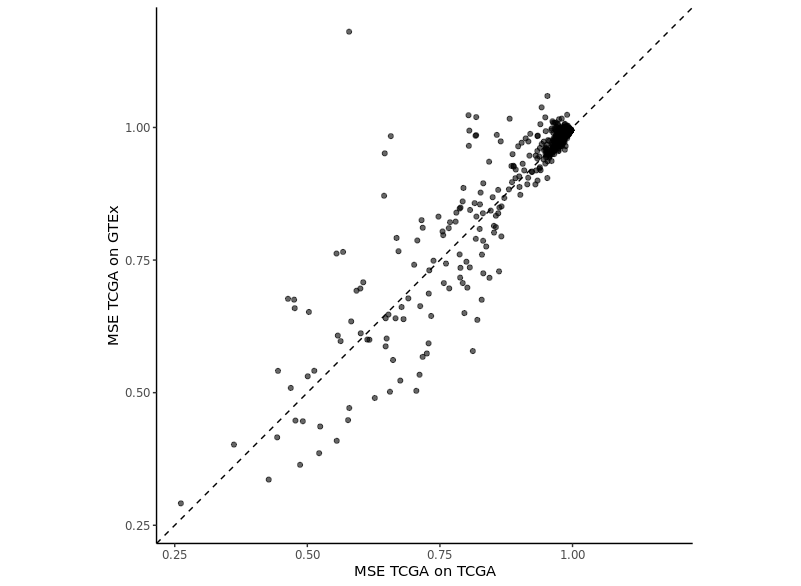
**


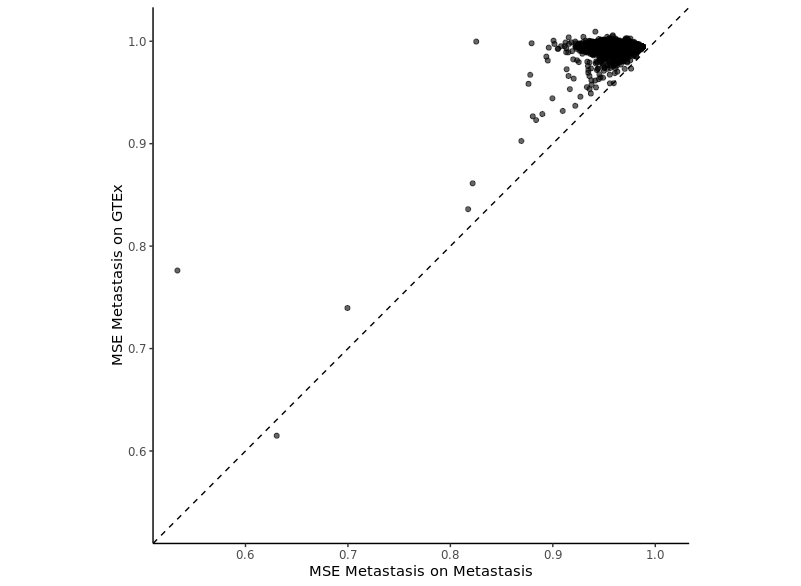
E) F)

**
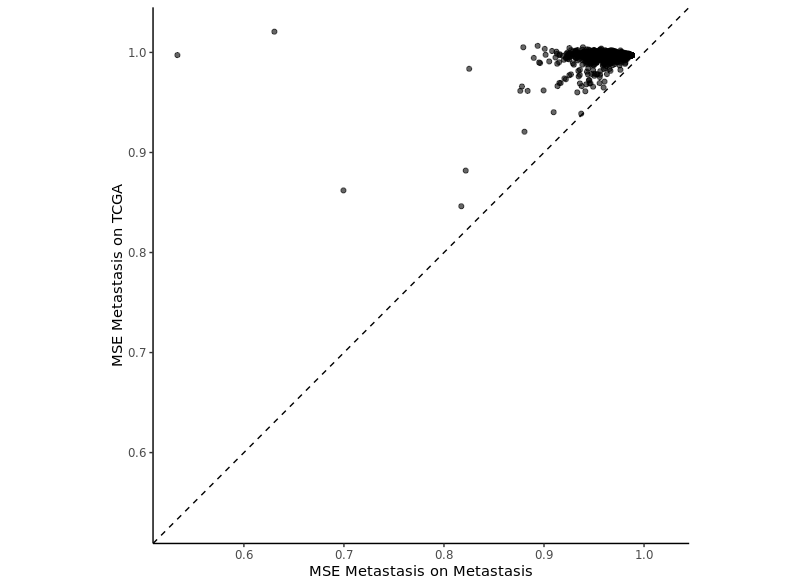
**

Figure S3: LD structure in the region of interest on chromosome 1. Pairwise LD, showing both r2 (red) and D’ (blue) is on the top. At the bottom is a schematic showing the position of all the genes in the region, with the significant genes highlighted in red. Note that the scale is based on SNP indexes and is therefore non-linear but monotonic with respect to genomic position.


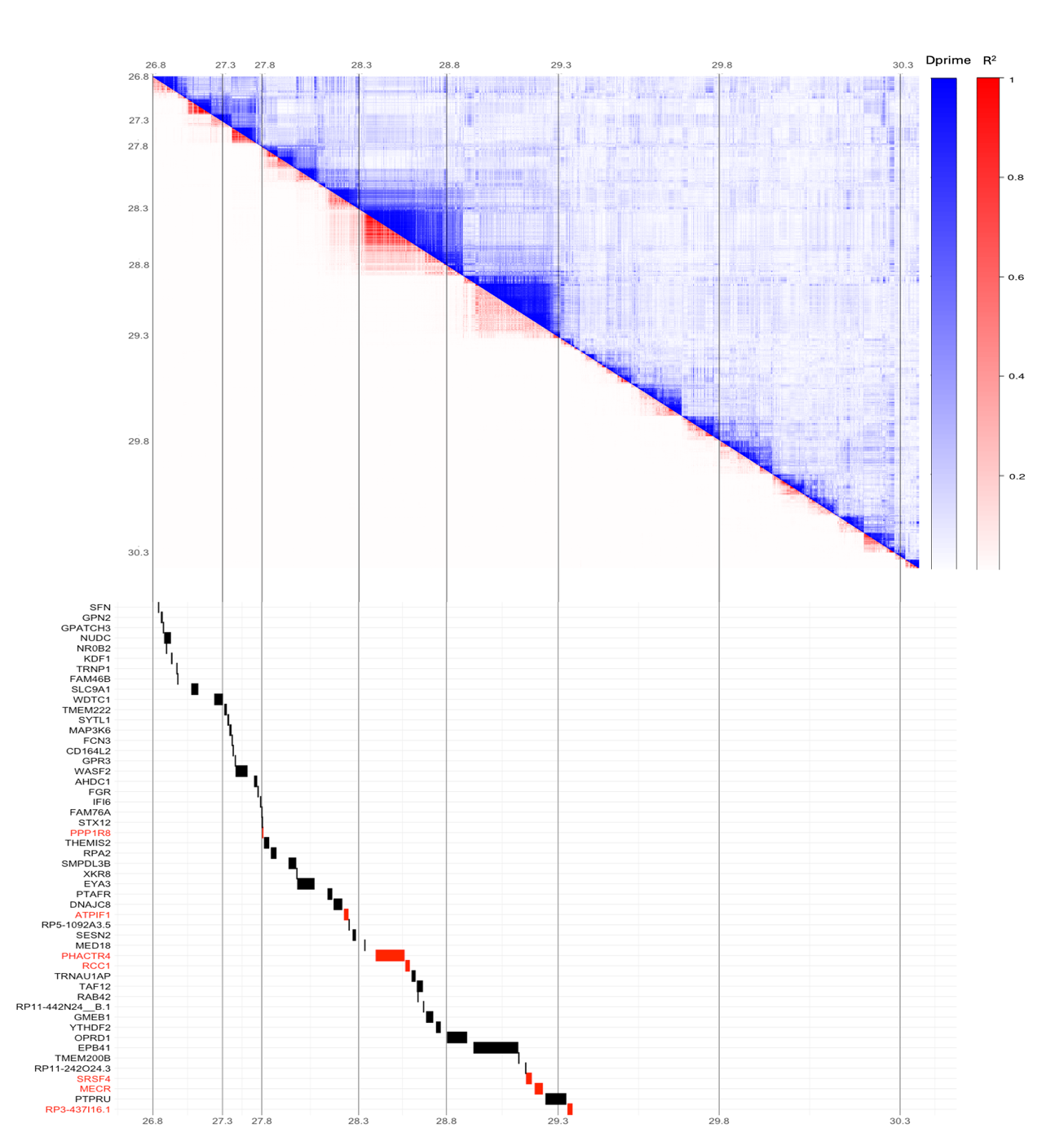


Figure S4 Correlation heatmap of measured expression, predicted expression in training data, predicted expression in PLCO for GTEx and metastasis

Table S2: Cross-validated R^2^ for PrediXcan (elastic net) model

| CVR^2^ | GTEx  (Normal) | TCGA  (Primary Tumor) | GCMCRPC (Metastasis) |
| --- | --- | --- | --- |
| <0.005 | 14862 (56%) | 16625 (74%) | 16430 (58%) |
| [0.005, 0.01) | 2616 (10%) | 2269 (10%) | 1788 (6%) |
| [0.01, 0.05) | 5497 (21%) | 2570 (11%) | 7332 (26%) |
| [0.05-0.1) | 1370 (5%) | 515 (2%) | 2076 (7%) |
| ≥0.1 | 2240 (8%) | 495 (2%) | 846 (3%) |
| Total | 26585 | 22474 | 28472 |
